# A DegU-P and DegQ-Dependent Regulatory Pathway for the K-state in *Bacillus subtilis*

**DOI:** 10.3389/fmicb.2016.01868

**Published:** 2016-11-22

**Authors:** Mathieu Miras, David Dubnau

**Affiliations:** ^1^Public Health Research Institute Center, New Jersey Medical School, Rutgers University, NewarkNJ, USA; ^2^Laboratoire de Microbiologie et Génétique Moléculaires, Université de ToulouseToulouse, France

**Keywords:** K-state, competence, bistability, DegU, DegQ, ComK, strain domestication, *Bacillus subtilis*

## Abstract

The K-state in the model bacterium *Bacillus subtilis* is associated with transformability (competence) as well as with growth arrest and tolerance for antibiotics. Entry into the K-state is determined by the stochastic activation of the transcription factor ComK and occurs in about ∼15% of the population in domesticated strains. Although the upstream mechanisms that regulate the K-state have been intensively studied and are well understood, it has remained unexplained why undomesticated isolates of *B. subtilis* are poorly transformable compared to their domesticated counterparts. We show here that this is because fewer cells enter the K-state, suggesting that a regulatory pathway limiting entry to the K-state is missing in domesticated strains. We find that loss of this limitation is largely due to an inactivating point mutation in the promoter of *degQ*. The resulting low level of DegQ decreases the concentration of phosphorylated DegU, which leads to the de-repression of the *srfA* operon and ultimately to the stabilization of ComK. As a result, more cells reach the threshold concentration of ComK needed to activate the auto-regulatory loop at the *comK* promoter. In addition, we demonstrate that the activation of *srfA* transcription in undomesticated strains is transient, turning off abruptly as cells enter the stationary phase. Thus, the K-state and transformability are more transient and less frequently expressed in the undomesticated strains. This limitation is more extreme than appreciated from studies of domesticated strains. Selection has apparently limited both the frequency and the duration of the bistably expressed K-state in wild strains, likely because of the high cost of growth arrest associated with the K-state. Future modeling of K-state regulation and of the fitness advantages and costs of the K-state must take these features into account.

## Introduction

The transcription factor ComK (van Sinderen et al., 1995) directly activates more than 100 genes (Berka et al., 2002; Hamoen et al., 2002; Ogura et al., 2002). While about 20 of these mediate the uptake, processing and integration of exogenous DNA resulting in transformation (Burton and Dubnau, 2010), the roles of the remaining ∼80 genes are poorly understood. Because these genes are not needed for transformation (J. Hahn, unpublished) the expression of ComK must result in phenotypes beyond competence that presumably enhance fitness. In fact, ComK also induces a period of growth arrest during which the cells that express ComK exhibit antibiotic tolerance (Nester and Stocker, 1963; Haijema et al., 2001; Johnsen et al., 2009; Briley et al., 2011; Hahn et al., 2015; Yuksel et al., 2016). This persistent state has been called the K-state, to emphasize that ComK regulates more than competence for transformation (Berka et al., 2002).

A long recognized and remarkable feature of the K-state is that it is expressed in a minor fraction of a clonal population, in a more or less all or nothing fashion (Nester and Stocker, 1963; Singh and Pitale, 1967; Hadden and Nester, 1968; Haseltine-Cahn and Fox, 1968; Maamar and Dubnau, 2005; Smits et al., 2005). Entry into the K-state is determined stochastically and studies in domesticated strains derived from the indole-requiring auxotrophic strain 168 (Spizizen, 1958) have ascribed this stochastic determination to noise in the basal expression of the *comK* promoter (Maamar et al., 2007; Süel et al., 2007). When the noisy expression of *comK* causes a subpopulation of cells to exceed a threshold level of ComK, two dimers of this protein bind cooperatively to the *comK* promoter (van Sinderen and Venema, 1994; Hamoen et al., 1998), activating a positive feedback loop and the rapid transition of these cells into the K-state, where ComK activates downstream genes.

The frequency of these activation events is extremely low during exponential growth and then rises as a culture approaches the stationary phase of growth. This temporal control has two principal causes. First, growing cultures secrete ComX, a quorum sensing pheromone, which accumulates and ultimately causes the phosphorylation of the response-regulator protein ComA (Magnuson et al., 1994). ComA-P then binds to and activates the promoter of the *srfA* operon, which encodes the small protein ComS (Nakano and Zuber, 1991; Nakano et al., 1991; Roggiani and Dubnau, 1993; Hamoen et al., 1995). ComS in turn competes with ComK for binding to the MecA-ClpC-ClpP protease, which rapidly degrades ComK during growth (Turgay et al., 1997, 1998; Prepiak and Dubnau, 2007). Stabilization occurs toward the end of exponential growth when high cell densities have produced sufficient ComX levels. A second cause of temporal regulation derives from the exquisitely controlled kinetics of *comK* basal expression (Leisner et al., 2007; Mirouze et al., 2012). Ensemble measurements show that the average basal rate of *comK* transcription increases gradually during growth, reaches a maximum as cells depart from exponential growth (T_0_), and then declines. This uptick in basal expression is due to a gradual increase in the phosphorylated form of the master regulator Spo0A as cells approach stationary phase (Mirouze et al., 2012). Spo0A is phosphorylated as a consequence of a phosphorylation cascade in which several kinases transfer phosphoryl groups to Spo0F (Burbulys et al., 1991). These groups are then passed to the phosphotransfer protein Spo0B and finally to Spo0A. Low levels of Spo0A-P directly activate the basal expression of *comK*, while higher levels bind to repressive operator sites so that the rate of *comK* expression decreases (Mirouze et al., 2012). Thus, Spo0A-P opens and then closes a temporal gate for transitions to the K-state.

Although K-state regulation has been well characterized, there was reason to believe that our understanding was lacking. It has been observed that the transformability of undomesticated isolates of *Bacillus subtilis* and its close relatives is much lower than that of the domesticated derivatives of 168 (Cohan et al., 1991). In fact the model undomesticated isolate NCIB3610 (hereafter 3610), is poorly transformable, although it is very closely related to the wild parent of 168. The poor transformability is due in part to *comI*, a gene that is absent in 168-derivatives. Interestingly, the ComI protein appears to decrease the uptake of DNA, without affecting K-state expression (Konkol et al., 2013). Together, these observations suggest that some regulatory feature has been lost in the domesticated strains and that our appreciation of K-state regulation is consequently incomplete.

In the present study, we have shown that a known promoter mutation in *degQ* in domesticated strains (Yang et al., 1986; McLoon et al., 2011) is primarily responsible for this difference in transformability. It is of interest that this mutation also contributes to the failure of strain 168 derivatives to form robust biofilms (McLoon et al., 2011). A consequence of this mutation is that the response regulator DegU is poorly phosphorylated in domesticated strains (Stanley and Lazazzera, 2005; Kobayashi, 2007). This deficit in DegU-P derepresses P*srfA*. This causes ComK to be stabilized, allowing more cells to pass the threshold for *comK* auto-activation, thereby increasing the fraction of K-state cells. It is known that unphosphorylated DegU is required for K-state expression because it helps ComK bind to its own promoter, thus acting as a priming protein when the ComK concentration is low (Hamoen et al., 2000). Thus, our present results show that the regulation of the K-state in undomesticated strains requires the proper ratio of phosphorylated and unphosphorylated DegU, in accordance with the view that this protein acts as a rheostat for development (Verhamme et al., 2007).

## Materials and Methods

### Microbiological Methods

Bacterial strains are listed in Supplementary Table S1. The backgrounds used for all experiments were either IS75, a derivative of strain 168, PS216 (an undomesticated strain of *B. subtilis* isolated in Slovenia and kindly provided by Inés Mandic-Mulec) (Durrett et al., 2013), 3610△*comI* or 3610 *comI^Q12L^* (both gifts from Dan Kearns). The *comI^Q12L^* mutation abolishes ComI activity (Konkol et al., 2013), removing a block in DNA uptake. Constructs were introduced into IS75 by transformation (Albano et al., 1987) and into 3610△*comI* and PS216 by transduction using bacteriophage SPP1 (Cozy and Kearns, 2010). An exception was for the swapping of the *degQ* alleles, which was carried out by transformation, as described below. Bacterial growth was at 37°C in competence medium (Albano et al., 1987) unless otherwise specified. Antibiotic selections were carried out on Lysogeny Broth (LB) agar plates (Cozy and Kearns, 2010) containing ampicillin (100 μg ml^-1^), spectinomycin (100 μg ml^-1^), erythromycin (5 μg ml^-1^), kanamycin (Kan) (5 μg ml^-1^) or chloramphenicol (5 μg ml^-1^). In some cases selection was for erythromycin (Ery) (1 μg ml^-1^) plus lincomycin (20 μg ml^-1^). Solid media were solidified by the addition of 1.5% agar. Transformation frequencies were determined using genomic DNA isolated from a leucine prototroph.

### Luciferase Assays

Light output from luciferase reporter constructs was measured as previously described (Mirouze et al., 2011). Briefly, strains were first grown in LB medium for 2 h. Cells were then centrifuged and resuspended in fresh competence medium (Albano et al., 1987), adjusting all the cultures to an OD_600_ of 2. These pre-cultures were then diluted 20-fold in fresh competence medium and 200 μl was distributed in duplicate in the wells of a 96-well black plate (Corning Incorporated Costar). Ten microliters of luciferin was added to each well to reach a final concentration of 1.5 mg/ml (4.7 mM). The cultures were incubated at 37°C with agitation in a PerkinElmer Envision 2104 Multilabel Reader equipped with an enhanced sensitivity photomultiplier for luminometry. The temperature of the clear plastic lid was maintained at 38°C to avoid condensation. Relative Luminescence Units (RLU) and OD_600_ were measured at 1 min intervals after two 30-s shaking steps. The data were processed using a script written in MATLAB, exported to Excel and plotted as RLU/OD versus time from the beginning of growth.

### SDS-PAGE and Immunoblotting

Cell pellets were resuspended in STM buffer (50 mM Tris pH 8.0, 25% sucrose, 50 mM NaCl, 5 mM MgCl_2_) containing 300 μg ml^-1^ lysozyme and incubated at 37°C for 5 min. The volume of STM was normalized to the turbidity measurement of the culture (determined in a Klett colorimeter) when the sample was collected. Sample buffer (final concentration of 20 mM Tris HCl pH 6.8, 10% glycerol, 1% SDS, 0.01% bromophenol blue, 2% 2-mercaptoethanol) was added to the samples, which were then incubated at 100°C for 5 min. Samples were separated by electrophoresis in 12% Tris-tricine SDS polyacrylamide gels (Schägger and von Jagow, 1987). The proteins were transferred to nitrocellulose membranes (Millipore) using a Trans Blot Turbo semidry transfer apparatus (Bio-Rad). Primary antiserum raised in rabbits against ComK was used at a dilution of 1:5000. Signal was detected using a secondary dilution of 1:10,000 goat anti-rabbit antiserum conjugated to horseradish-peroxidase (HRP) followed by visualization using Enhanced Chemiluminescence (ECL) Prime Western Blot Detection Reagent (GE Healthcare) according to the manufacturers’ instructions. Images were recorded by using a Thermo Scientific MyECL Imager and band intensities were measured using ImageJ software (Schneider et al., 2012).

### *degQ* Allele Swapping with pMiniMAD2

To swap the *degQ* promoter between IS75 and 3610 at the native loci, we used the pMiniMAD2 cloning strategy as described (Cozy and Kearns, 2010; Mukherjee et al., 2013). A 2 kb fragment containing *degQ* from strain 168 or 3610 was amplified using primers (HinDIII-degQ9: 5′-GCAGCAAAGCTTCTGCGATTTCCGGATAAAAGAACATAATAATCCCAG-3′ and BamHI-degQ10: 5′-GCAGCAGGATCCGCAGCCTGCTTCTTATATGCTGATCG-3′). The amplicons, which carried the *degQ* wild and mutant alleles near their centers, were cloned into the *HinD*III and *BamH*I sites on pMiniMAD2, to produce the plasmids pED1932 and pED1931, which carried the wild-type and mutant *degQ* alleles, respectively. These plasmids were used to transform IS75 (with pED1932) and 3610 (with pED1931) where they integrated by single crossover events. Plasmid-free strains carrying the swapped *degQ* promoters were isolated (Cozy and Kearns, 2010; Mukherjee et al., 2013) to create 168 *degQ^3610^* (BD7454) and 3610 *degQ^IS75^* (BD7445). The presence of the swapped promoters was confirmed by sequencing of PCR products amplified from the chromosome, carried out by Eton Biosciences (Union, NJ, USA).

### The P*hyper-spank*-*degQ* Construction

The primers degQ15 (5′-TTAGTCGACAGCTAGCCACCATACACAATTCATTGATCTTTCA-3′) and degQ16 (5′-CTTGCATGCGGCTAGCTACTCGTTAATCCTACTGTATACAAGGA-3′) were used to amplify a 676 bp sequence containing the *degQ* gene without its promoter. The amplicon was inserted into the P*hyper-spank* vector pED1870 that had been cut with *Nhe*I, using the In-Fusion HD cloning kit (Clontech, Inc.), as per the manufacturer’s instructions. The resulting plasmid was integrated into the desired host strains by transformation with single crossover events, by selection for Kan resistance. pED1870 carries the P*hyper-spank, lacI* and *lacO* sequences. This and all other constructs were verified by sequencing. The resulting plasmid creates a strain in which *degQ* is under P*hyper-spank* at its native locus.

### Microscopy

Cells were harvested, diluted into PBS (81 mM Na_2_HPO_4_ + 24.6 mM NaH_2_PO_4_ + 100 mM NaCl) and 1 μl of each culture was placed on a pad of 1% agarose made up in 0.5X TAE buffer. Images were collected using a Nikon Eclipse Ti inverted microscope equipped with an Orca Flash 4.0 digital camera (Hamamatsu), with a Nikon TIRF 1.45 NA Plan Neoflur 100 oil immersion objective. NIS-Elements AR (v 4.40, Nikon) software was used to collect and analyze images, which were then imported into Photoshop to configure the images for publication. Fluorescence intensities were determined using the automated General Analysis tool of NIS-Elements.

## Results

### Undomesticated *Bacillus subtilis* Strains Express the K-state in Few Cells

**FIGURE 1:**
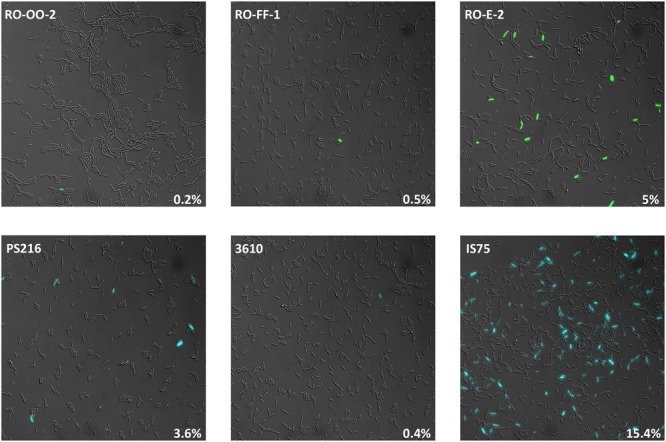
**Undomesticated strains express the K-state with low frequency.** Shown are representative images of the indicated strains expressing P*comK* fusions to the genes encoding GFP (top row) or CFP (bottom row). The fields were selected to show at least one expressing cell. The measured frequencies of K-state cells are indicated in the lower right of each panel. As explained in the text, the domesticated strain (IS75) was imaged at T_2_ and the undomesticated strains at T_0_. Strain numbers are presented in Supplementary Table S1.

It has been observed that the transformability of undomesticated *B. subtilis* strains is generally lower than that of the domesticated strain 168 and its derivatives (Cohan et al., 1991). Because the K-state is expressed bistably, this difference may reflect differing efficiencies of transformation per cell or differing frequencies of K-state cells in the population. We therefore determined the percentage of competence-expressing cells during growth in five randomly chosen natural isolates: the *B. subtilis* subsp. *subtilis* RO-OO-2 and RO-FF-1 strains, the *B. subtilis* subsp. *spizizenii* RO-E-2 [all three isolated in the Mojave desert (Cohan et al., 1991)], the commonly used model strain 3610 (Branda et al., 2006; McLoon et al., 2011) and *B. subtilis* PS216, isolated in Slovenia (Durrett et al., 2013). Throughout this study we have used *comI* mutants of 3610. ComI does not affect the expression of *comK* but does inhibit the uptake of transforming DNA (Konkol et al., 2013). In each case, after verifying that the regulatory sequences upstream of *comK* were the same in all the strains (not shown), *comK* promoter (P*comK*) fusions to the genes encoding Cyan Fluorescent Protein (CFP) or Green Fluorescent Protein (GFP) were integrated by single reciprocal recombination and the percentages of *comK-*expressing cells were enumerated microscopically and compared to that of the 168-derivative IS75, which is the reference domesticated strain used throughout this study. We determined that the time of maximal transformability for PS216 and 3610 was attained after 240 min of growth and after 315 min for IS75 (Supplementary Figure S1). The results in **Figure 1** were therefore obtained with samples taken at those times, which correspond to about T_0_ and T_2_ for the undomesticated and domesticated strains, respectively. The growth curves for all the strains under these conditions were similar (not shown). The results summarized in **Figure 1** show that the samples from the undomesticated isolates contain significantly fewer competence-expressing cells (0.2–5%) than the domesticated strain sample (15.4%).

**FIGURE 2:**
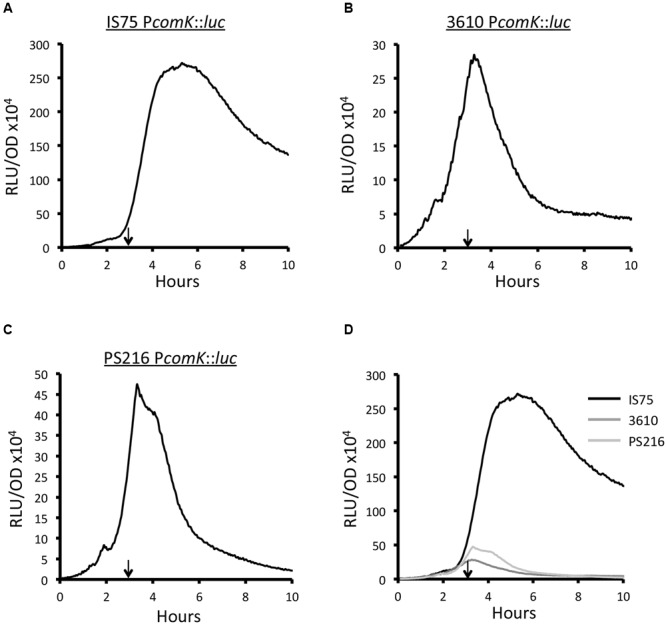
**Expression from P*comK* is higher in IS75 (A)** than in the undomesticated strains 3610 **(B)** and PS216 **(C)**. **(D)** The three curves plotted on the same scale. The vertical arrows on panels **(A–C)** point to T_0_. Strain numbers are presented in Supplementary Table S1.

To compare the transcription rates from P*comK* in strains 3610 and PS216 to that of the domesticated strain, we utilized promoter fusions to firefly luciferase, which reports transcription rates rather than the accumulation of a gene product (Mirouze et al., 2011). The fusions were integrated by single-reciprocal recombination placing the reporter gene under control of the normal *comK* regulatory sequences. The peak rates of transcription from P*comK* in the domesticated strain and in both 3610 and PS216 approximately reflect the frequencies of K-state cells in each population (**Figure 2**). The peak transcription rates in the undomesticated strains are about 9.6- and 5.7-fold lower than in the IS75 background, while the fractions of competence-expressing cells differ by 38- and 4.2-fold, respectively. We do not expect an exact correspondence between the peak rates of transcription and transformation frequencies; light output from luciferase does not inform us about the amount of ComK synthesized, the activation of the downstream genes needed for transformation and the assembly of the transformation machinery. In the domesticated strain, the transcription rate remains elevated even as the cultures enter stationary phase (**Figure 2A**). In 3610 and PS216, the rates decline rapidly from a maximum reached at about T_0_ (**Figures 2B,C**). These data demonstrate that unknown mechanisms limit the probability of transitions to the K-state and cause the expression of *comK* to cease abruptly. Interestingly, these mechanisms have been lost in the domesticated strains.

### A *degQ* Mutation in the Domesticated Strains Causes Increased *comK* Expression

To identify gene(s) that limit transitions to the K-state in undomesticated strains, we transformed PS216 with DNA from a domesticated strain that carries a fusion of the *comG* promoter to *lacZ*, linked to a kanamycin (Kan) resistance determinant. It was reasoned that selection for this marker on plates containing Kan and 5-bromo-4-chloro-3-indolyl-β-D-galactopyranoside (X-gal) would select for transformable cells and might transfer an unlinked mutation from the domesticated strain, capable of conferring increased expression from P*comG*. Indeed, dark blue colonies were observed, representing about 1% of the total number of Kan^R^ colonies, a frequency consistent with “congression,” the simultaneous transformation of *B. subtilis* by unlinked markers. Although PS216 colonies are normally mucoid, all of these blue colonies were not. Mucoidy in *B. subtilis* indicates the production of poly-γ-glutamic acid, due to the expression of the *pgs* operon and is dependent on the presence of phosphorylated DegU and the small protein, DegQ (Stanley and Lazazzera, 2005). DegQ increases the net transfer of a phosphoryl group from the histidine kinase DegS to its cognate response regulator protein DegU (Kobayashi, 2007). Because derivatives of strain 168 carry a promoter mutation that markedly decreases the expression of *degQ* (Yang et al., 1986; McLoon et al., 2011), we reasoned that the blue colonies might have inherited this mutation. The *degQ* promoters of several blue transformants were sequenced and indeed all had the T→C promoter mutation at position -10 that exists in 168 derivatives.

**FIGURE 3:**
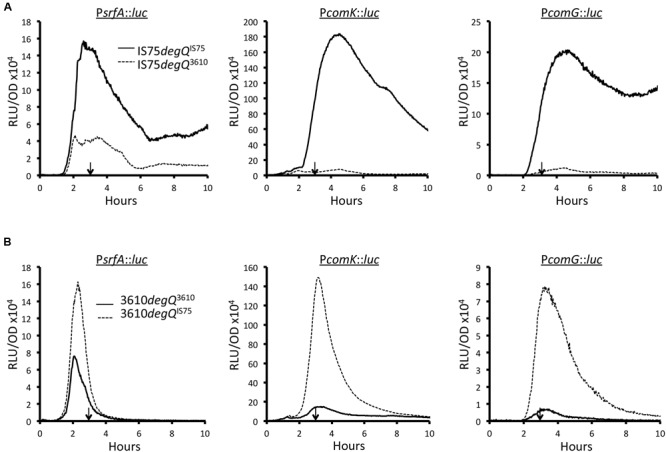
**Effect of the mutant (*degQ^IS75^*) and wild-type (*degQ^3610^*) *degQ* alleles on expression of *srfA, comK*, and *comG* in IS75 (A)** and 3610 **(B)** backgrounds. In each panel, the data from strains with indigenous and swapped alleles are indicated by solid and dashed lines, respectively. The vertical arrows in each panel point to T_0_. Strain numbers are presented in Supplementary Table S1.

To determine whether this mutation was responsible for the high *comK* expression of domesticated strains, we swapped the wild-type and mutant *degQ* alleles between the domesticated and undomesticated strains, using pMiniMAD2. For this, and for all subsequent experiments reported here, we have used 3610, which has been widely adopted as a model undomesticated *B. subtilis*, rather than the less intensively studied PS216. **Figure 3A** shows the expression profiles of P*srfA*, P*comK*, and P*comG* luciferase fusions in the IS75 background, for strains carrying the indigenous mutant (*degQ^IS75^*) and wild-type (*degQ^3610^*) alleles. Clearly the introduction of the wild-type *degQ* allele resulted in a marked diminution of the transcription rates of each reporter so that the peak levels were similar to those of 3610 (compare to **Figure 3B**). The converse was also true (**Figure 3B**); introduction of the *degQ^IS75^* allele into the 3610 background increased the maximum transcription rates of all three promoters to approximately the levels measured in IS75 (**Figure 3A**). A *degQ* knockout was also tested (Supplementary Figure S2) using a P*comG-luc* reporter in the 3610 background. As expected, a large increase in *comG* transcription was noted. The effects of *degQ* allele swapping shown in **Figure 3** can thus be explained most economically by a repressing effect of DegU-P on P*srfA*, which would decrease the amount of ComS, destabilizing ComK. In fact it has been shown that mutations resulting in an elevated level of DegU-P do indeed depress the transcription of *srfA* in the domesticated background (Hahn and Dubnau, 1991).

### The Sharp Decrease in Transcription in 3610 is Not Due to DegQ

Somewhat surprisingly, the characteristic sharp decreases in the transcription rates of *srfA* and of *comK* in the 3610 background are not affected by the introduction of the mutant *degQ* promoter (**Figure 3**). Nor are the sustained high transcription rates from these promoters in the domesticated background obliterated when the wild-type *degQ* allele is introduced. This is seen clearly when the peak values of the swapped and un-swapped strains are normalized (Supplementary Figure S3). The *comK* transcription patterns can be explained by differences in *srfA* transcription if we make the simplifying assumption that ComS is unstable. Thus, when the rate of *comS* transcription decreases sharply, ComK would no longer be protected from degradation, and the transcription of *comK* and of *comG* would decrease. In accord with this reasoning, the transcription rates of *srfA, comK* and *comG* begin to drop after 2.3, 3.1, and 3.1 h of growth, respectively (**Figure 3**). The converse would apply in the domesticated strains; sustained transcription from the *srfA* promoter would provide a steady supply of ComS, stabilizing ComK. To test this idea, we measured the transcription rates of *srfA* in three different backgrounds. As noted above, PS216 is intermediate between 3610 and IS75 in transformability, in *comK* expression and in the percentage of cells that enter the K-state. Supplementary Figure S4 shows that while transcription from P*srfA* drops to zero in 3610, it decreases to an intermediate level in PS216. This can be seen clearly when the three curves are normalized to the same peak value (Supplementary Figure S4B). These comparisons are in agreement with the hypothesis that the kinetics of *srfA* transcription underlie the strain differences in transformability.

### The DegQ Effect on K-state Expression Is Mediated by Increased DegU-P

**FIGURE 4:**
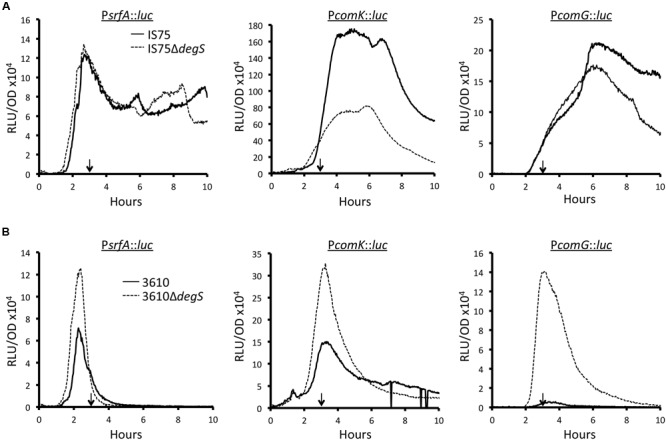
**Effect of *ΔdegS* on expression of *srfA, comK*, and *comG* in IS75 (A)** and 3610 **(B)** backgrounds. The vertical arrows in each panel point to T_0_. Strain numbers are presented in Supplementary Table S1.

If the presence of the mutant *degQ* allele affects competence expression because of its depressing effect on DegU phosphorylation, it should be mimicked, at least in 3610, by inactivation of *degS*, which encodes the DegU-cognate histidine kinase. The results obtained in the 3610 background support a role for DegU-P as a regulator of *srfA* and hence of *comK* and *comG* expression (**Figure 4**). The *degS* knockout strain reaches a higher rate of *srfA* transcription than the wild-type and presumably produces more ComS (**Figure 4B**). Consequently ComK is stabilized, more *comK* transcription takes place and *comG* transcription is actually increased (**Figure 4B**). In contrast with this straightforward result, **Figure 4A** reveals that the inactivation of *degS* has no effect on *srfA* transcription in the IS75 background. This is not unexpected, because in this background, which is naturally lacking in DegQ, the amount of DegU-P is low and does not limit *srfA* transcription. However, the expression from *comK* is affected, decreasing in peak value about twofold. This relatively minor effect of *degS* inactivation can be explained as follows. Unphosphorylated DegU is known to increase the affinity of ComK for its own promoter (Hamoen et al., 2000). Because DegU-P activates one of the promoters that drive *degU* expression (Ogura and Tsukahara, 2010), it is likely that the inactivation of *degS* decreases the amount of DegU and thus compromises ComK binding to P*comK*. The expression from P*comG* is also affected (**Figure 4A**) but less than that from P*comK*, suggesting that ComK is normally produced in excess.

Further evidence that the depressing effect on *srfA* expression in 3610 is due to phosphorylated DegU was obtained using a *degUD56N* allele. The DegU^D56N^ protein cannot be phosphorylated. Supplementary Figure S5 shows that the DegU^D56N^ mutant strain is de-repressed for *srfA* transcription similarly to the *degS* inactivated strain (compare to **Figure 4B**). As observed when the *degQ* alleles were swapped (**Figure 3**; Supplementary Figure S3), no effect of either the D56N or *degS* knockout mutations was observed on the sharp downturn in *srfA* transcription in the 3610 background.

### DegQ Regulates the Stability of ComK

**FIGURE 5:**
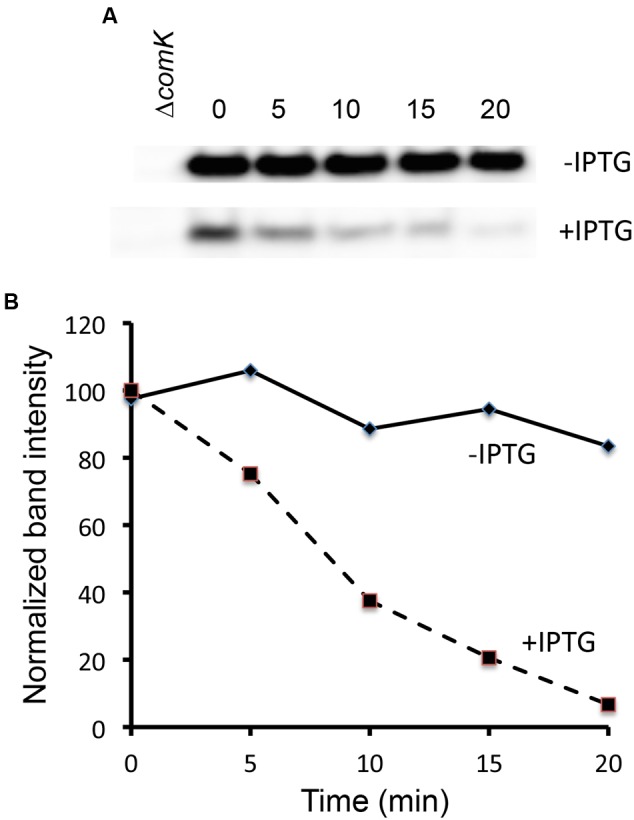
**DegQ lowers ComK stability.** A strain carrying P*xyl-comK* and P*hyper-spank-degQ* (BD8288) was grown in the presence of xylose and with and without IPTG. At T_-1_, puromycin and rifampicin, each at 200 μg/ml were added. At the indicated times, samples were withdrawn for Western blotting with anti-ComK antiserum. **(A)** The -IPTG gel was exposed for a shorter time to compensate for its higher initial intensity of the ComK band. Extracts from a *ΔcomK* strain were included to assist in the identification of the correct band. The ±IPTG images were from the same gel and placed one above the other **(B)** Bands intensities were quantified from digitized images using ImageJ software and plotted against time after the addition of the inhibitors.

If the effects of DegU-P on P*srfA*, and hence on *comS* transcription underlie the differences in K-state expression, we would expect to see this reflected in measurements of ComK stability. Because the amount of ComK in the presence of elevated DegQ is quite low, we resorted to the following strategy to investigate its stability. A strain was constructed that carries a copy of *comK* under control of the xylose-inducible P*xyl* promoter inserted in the ectopic *amyE* locus of IS75. The native *comK* gene was left intact so that the effects of induction due to the addition of xylose would be amplified by the auto-activation of *comK* transcription. Also present was a copy of *degQ* under control of the isopropyl-β-D-thiogalactoside (IPTG)-inducible P*hyper-spank* promoter, located in the chromosome at the *degQ* locus. This strain was grown in the continuous presence of xylose, with and without IPTG. When late log phase was reached, rifampicin and puromycin were added to terminate mRNA and protein synthesis, samples were collected thereafter at regular intervals, and Western blotting was used to monitor the decay of ComK (**Figure 5**). As predicted, the stability of ComK was markedly reduced when *degQ* was induced. In the uninduced culture the half-life of ComK is well in excess of 20 min; extrapolation would suggest it to be at least 60 min. The half-life of ComK in the presence of induced DegQ is 7–8 min. As expected, the initial amount of ComK was also reduced in the induced culture. These data confirm that the enhanced transformability of the domesticated strain can be ascribed to the increased stability of ComK, caused by the de-repression of *srfA* (*comS*).

### Single Cell Expression in *degQ*-Swapped Strains

**FIGURE 6:**
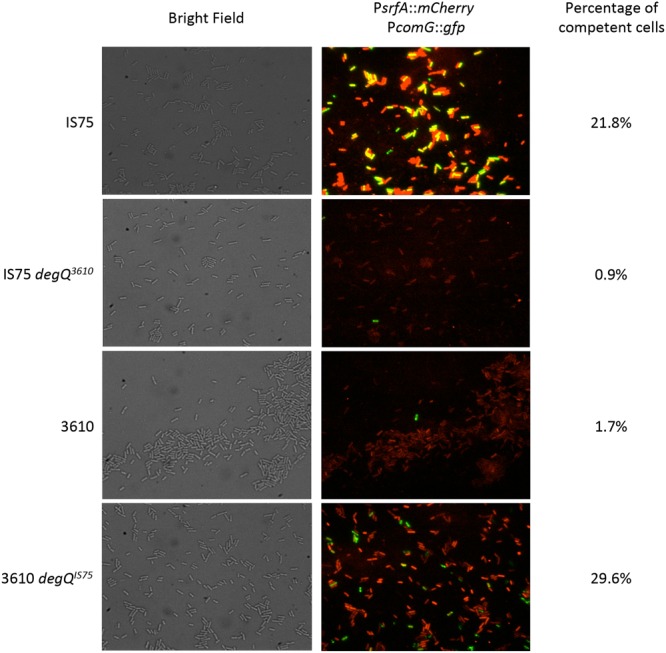
**Single-cell expression of P*srfA-mCherry* and P*comG-gfp*.** The indicated strains, all of which carried these two fusion constructs, were grown to the time of maximum K-state expression and samples were taken for microscopy. Representative images are shown. The 3610 and IS75 degQ^3610^ images were selected to include at least one K-state cell each. On the right are the percentages of K-state cells determined by examining at least 1200 cells for each strain. Strain numbers are presented in Supplementary Table S1.

Ensemble measurements of *srfA* and *comG* transcription show that their expression is correlated, and influenced by the levels of DegU-P. **Figure 6** shows this behavior on a single cell level. For these experiments, cells expressing promoter fusions of genes encoding mCherry and GFP to P*srfA* and P*comG*, respectively, were examined microscopically. Unlike firefly luciferase these reporters reflect the accumulation of gene products. These images were collected for the wild-type IS75 and 3610 strains as well as for strains in which the *degQ* alleles had been swapped. It is obvious that the GFP expressed from P*comG* accumulated in more cells in the IS75 background than in 3610, as expected. Also as expected, the intensity of the mCherry fluorescence expressed from P*srfA* was much higher in IS75 than in the undomesticated strain. When the *degQ^IS75^* allele was introduced into 3610, the expression from P*srfA* increased and the frequency of P*comG*-expressing cells also increased. Nevertheless, the mCherry fluorescence in IS75 is still more intense than that of the 3610 *degQ^IS75^* strain, despite the fact that the maximum rates of *srfA* expression are the same in both strains (**Figure 3**). This probably reflects the sustained *srfA* transcription rate in IS75 and the sharp decrease in the 3610 background. As expected, when *degQ^3610^* was swapped into IS75 the mCherry fluorescence decreased to a level lower than that of wild-type IS75 and even lower than that of 3610 *degQ^IS75^*, consistent with the fourfold higher amplitude of the P*srfA* transcription rate curve in 3610 *degQ^IS75^* than in IS75 *degQ^3610^* (**Figure 3**). These data demonstrate that the results presented above for promoter transcription rates were reflected on the single-cell level.

We next sought to determine whether the K-state was expressed preferentially in cells that had accumulated more mCherry, expressed from P*srfA*. For this, we measured the average pixel intensities of mCherry in K-state cells, identified by their GFP signals, compared to the intensities of non-K-state cells. Equal numbers of K-state and non-K-state cells were selected from each microscope field to minimize inter-field bias. **Table 1** shows that at the time of these measurements, K-state cells in both the 3610 and IS75 backgrounds do not appear to exhibit a noticeably different mCherry signal than the non-K-state cells. This would suggest that variation in transcription from P*srfA* may not be an important determinant of K-state transitions. However, this conclusion must be tempered by two considerations. First, the decision to enter the K-state was made, on the average, before these measurements were made. Second, the mCherry signal does not necessarily reflect the concentration of ComS.

**Table 1:** Accumulation of signal from P*srfA-mCherry*^1^.

Strain	Strain Background	K-state	Non-K-state
	3610	59 ± 14	55 ± 11
	IS75	243 ± 31	306 ± 43

*^1^Average pixel intensities and standard deviations were calculated for each strain after background subtractions.*

### RapP Influences the Sharp Downturn in *srfA* Expression in 3610

As noted above (**Figure 3**), the sharp downturn in *srfA*, and hence in *comK* and *comG* transcription, is clearly not dependent on DegQ and remains unexplained. We have considered the possibility that the differing kinetics of *srfA* transcription in 3610 and IS75 are influenced by the absence of a functional copy of *swrA* in 168-derivatives, because SwrA has been reported to modulate the binding of DegU-P to some promoters (Ogura and Tsukahara, 2012). To test this we used pMiniMAD2 to swap the functional and mutant *swrA* alleles between the two strains. No effect on *srfA* transcription was observed (not shown). Similarly, we wondered whether surfactin, the product of the *srfA* operon, would exert an effect on the kinetics of *srfA* transcription. Accordingly, we inactivated the *srfA* operon in the 3610 strain and no effect on P*srfA* transcription was observed (not shown). Another strain difference is that 3610 carries a large plasmid, which encodes RapP, a phosphatase that acts on Spo0F-P (Parashar et al., 2013). It has been reported that the inactivation of *rapP* mitigates the sharp downturn in *srfA* transcription (Parashar et al., 2013; Omer Bendori et al., 2015). This result has been verified in our hands; we have consistently observed a shoulder in the *srfA* transcription rate curves in the *rapP* knockout strain, and we have found that the transcription rate does not drop to zero as it does in the *rapP^+^* parent (Supplementary Figure S6A). Although the inactivation of *rapP* mitigates the downturn in *srfA* transcription in 3610, it does not phenocopy the sustained transcription observed in IS75 (compare Supplementary Figure S6A with **Figure 4A**). Clearly other genes must be involved that differ between the domesticated and undomesticated strains.

Given the more sustained transcription of *srfA* in the 3610 *ΔrapP* strain, we would expect the transcription of *comK* to increase due to stabilization of ComK. Instead, the transcription of *comK* was reduced (Supplementary Figure S6B). This unexpected effect was caused by a depressing effect of *rapP* inactivation on the basal level of *comK* transcription. This is shown by the expression of P*comK* when auto-regulation is abrogated due to the absence of a functional copy of *comK* (Supplementary Figure S6C). In the *comK rapP* background, the basal expression of *comK* first increases as in the *rapP^+^* strain but then abruptly declines.

These data have an interesting implication; although PS216, like IS75, lacks a *rapP* ortholog, the basal level expression of *comK* is similar in amplitude and overall kinetics in IS75, 3610, and PS216 (Supplementary Figure S7). It appears that selection has used both RapP-dependent and independent mechanisms to maintain the balanced increase and decrease in basal *comK* expression.

## Discussion

**FIGURE 7:**
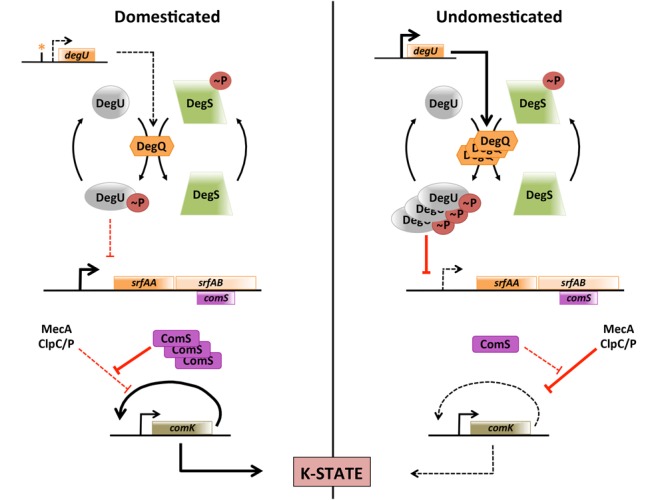
**K-state regulation in domesticated and undomesticated strains.** Dotted lines indicate weak or absent processes. In domesticated *B. subtilis* a promoter mutation decreases the rate of *degQ* transcription. The resulting low level of DegQ decreases DegU phosphorylation, relieving repression at the *srfA* promoter. As a result more ComS is synthesized and the degradation of ComK by MecA/ClpCP is decreased. It is not known whether the effect of DegU-P on *srfA* transcription is mediated by direct binding to the *srfA* promoter.

The first important conclusion of this study is that the frequency of K-state cells in the population is controlled in natural isolates by a pathway that regulates the amount of DegU-P, providing one more illustration of the importance of using undomesticated strains as a way to approximate real-life biology (McLoon et al., 2011). This previously unrecognized pathway for K-state regulation, summarized in **Figure 7**, acts by controlling the transcription of the *srfA* operon, which in turn affects the stability of ComK. Although noise in the basal expression of *comK* selects cells for competence, it appears that the instability of ComK in undomesticated strains effectively decreases the fraction of cells that exceed the threshold level of ComK needed to activate the auto-induction of *comK* transcription. This pathway is lost in 168-derivatives due to a promoter mutation that reduces the transcription of *degQ*. Since this mutation is present in all of the sequenced 168 derivatives, it must have been present in 168 itself, and is responsible for the choice of the indole-requiring 168 strain as a highly transformable subject for further investigation (Spizizen, 1958). We do not know how the elevated level of DegU-P that is present in 3610 acts to decrease the transcription from P*srfA*, nor do we know whether DegU-P acts directly on this promoter. Although *degS* and *degU* mutations that cause the accumulation of very high levels of DegU-P were known to inhibit the K-state (Msadek et al., 1990; Hahn and Dubnau, 1991), we show here for the first time that this mechanism is biologically relevant; DegU plays both positive and negative roles in regulation of the K-state in undomesticated strains. It is conceivable that the mucoidy, which can accompany increased DegU-P may itself compromise the binding of DNA to K-state cells, thereby contributing to the low transformability of undomesticated strains apart from the effect of DegU-P on P*srfA*.

Interestingly, P*degQ* is activated by ComA-P (Msadek et al., 1991), suggesting the following attractive mechanism. As the pheromone ComX accumulates, both *comS* and *degQ* would be transcribed. ComS would then stabilize ComK, helping to activate the auto-regulation of *comK* transcription, while DegQ would accumulate, increasing the concentration of DegU-P and eventually shutting down *srfA* transcription. However, as shown above (**Figure 3B**), the sharp down turn in *srfA* transcription that occurs in 3610 is not dependent on DegQ. Instead it is partly dependent on RapP, a phosphatase that acts on Spo0F-P, presumably restricting the rate of Spo0A-P accumulation. We conclude that this pleasing model does not seem to be true, at least under laboratory conditions.

Unphosphorylated DegU binds to sequences upstream from *comK*, helping ComK bind to its own promoter (Hamoen et al., 2000). We have shown here that DegU-P plays an important role in 3610, PS216 and presumably in other natural isolates, restricting the expression of *srfA* and hence of *comK*. Because DegU and DegU-P have respective positive and negative effects on K-state expression, the ratio of their concentrations must be critically controlled. Because *srfA* is essential for biofilm formation (Lopez et al., 2009), swarming motility (Kearns et al., 2004) and surface spreading (Kinsinger et al., 2003), the balance of phosphorylated and unphosphorylated DegU is also important for these forms of development, beyond the role of DegU-P in activating the expression of the hydrophobin BslA for biofilms (Hobley et al., 2013). In particular, swarming requires DegU-P (Verhamme et al., 2007). Biofilm formation is likewise inhibited when the amount of DegU-P is too high (Kobayashi, 2007; Verhamme et al., 2007). The mechanisms and upstream signals that control the level of DegU phosphorylation are not clear, but are certainly complex (Jers et al., 2011; Cairns et al., 2015) and important to elucidate.

As described above, Spo0A phosphorylation sets a temporal gate, opening and closing a window of opportunity for transitions to the K-state. Although phosphorylated and unphosphorylated DegU also play both positive and negative roles in the K-state, they may not control a temporal gate, because neither the inactivation of *degS* (**Figure 4B**) nor introduction of the non-phosphorylatable mutant form of DegU (D56N) (Supplementary Figure S5) affect the sharp downturn in *srfA* transcription in 3610. Perhaps instead, the ratio of DegU and DegU-P is simply adjusted in response to upstream signals to help set the probability of K-state development.

The inactivation of the RapP phosphatase in 3610 markedly decreases the basal expression from P*comK*, thus reducing transitions to the K-state (Supplementary Figure S6). It is possible that RapP has an activity aside from its ability to dephosphorylate Spo0F-P and that an unknown gene in this strain down-regulates the basal transcription of *comK* in the absence of RapP. However, because Spo0A-P is known to repress the basal expression of *comK*, it seems more likely that in the absence of RapP, excess phosphorelay activity increases the production of Spo0A-P and represses P*comK*. This is consistent with the kinetics of the basal expression shown in Supplementary Figure S7, which displays an initial rise identical to that in the *rap^+^* strain, followed by sharp repression. However, regardless of the presence or absence of *rapP*, the basal expression of *comK* in IS75, PS216, and 3610 are similar (Supplementary Figure S7). Apparently, additional factors in IS75 and PS216 must serve to control the phosphorelay in the absence of RapP. Selection, whether in the laboratory or in nature, precisely modulates the formation of Spo0A-P, probably because of the multiple and dramatic consequences imposed by the presence of too much or too little of this key transcription factor. We have shown elsewhere that 168-derivatives carry a mutation in *sigH*, which decreases the activity of the phosphorelay compared to that of 3610 (Dubnau et al., 2016). Using pMiniMAD2, we have swapped *sigH^168^* into a 3610 derivative lacking *rapP* and found that the basal level of *comK* transcription is still reduced (not shown). We suggest that some other difference between IS75 and 3610 is responsible for retarding Spo0A-P accumulation under K-state conditions.

*Bacillus subtilis* K-state cells are growth-arrested (Nester and Stocker, 1963; Haijema et al., 2001). The K-state appears to be a persistent state in which a trade off between the costs of growth-arrest and the expression of the competence machinery are balanced by fitness benefits due to tolerance in the face of environmental insults, e.g., exposure to antibiotics (Nester and Stocker, 1963; Johnsen et al., 2009; Hahn et al., 2015; Yuksel et al., 2016), as well as the ability to acquire useful genetic information. In the face of this trade-off, bet-hedging due to the bistable expression of the K-state, presumably helps to maximize fitness (Dubnau and Losick, 2006; Veening et al., 2008). It seems reasonable to assume that the frequency of K-state expression must be adjusted to obtain the biggest fitness advantage. Selection appears to have set the basal expression of *comK* and the kinetics of *srfA* expression to keep the frequency and timing of transitions to the K-state near an optimum. As noted above it has done this by at least two distinct mechanisms; RapP-dependent and RapP-independent. What appear to be important for fitness are the final kinetic behaviors of *srfA* and of *comK* basal expression. Although the transition frequency varies among undomesticated strains, it appears to be lower than in 168-derivatives, suggesting that the optimal bet-hedging frequency lies in a range below ∼15% (**Figure 1**).

Not only do the expressions of *srfA, comK*, and *comG* decline abruptly in PS216 and 3610, but so does transformability (Supplementary Figure S1). In other words competence, as traditionally defined, is restricted in these two strains to the time of entry to stationary phase, reaching a maximum at about T_0_ and decreasing thereafter. Perhaps this rapid decrease is due to the turnover of competence proteins, which might provide resources for the eventual growth of cells that are hunkered down in a persistent state. ComK expression is not terminated abruptly in IS75, so this turnover might have been overlooked. This would imply the existence of an additional K-state-related mechanism that has been lost during domestication, masking the natural transience of transformability.

## Author Contributions

Both MM and DD contributed to the planning and interpretation of experiments and to the writing of this manuscript. MM carried out the experimental work.

## Conflict of Interest Statement

The authors declare that the research was conducted in the absence of any commercial or financial relationships that could be construed as a potential conflict of interest.
