# Supplementary material for: A DegU-P and DegQ-Dependent Regulatory Pathway for the K-state in Bacillus subtilis
Source: Front Microbiol. 2016 Nov 22;7:1868. doi: 10.3389/fmicb.2016.01868 (PMC5118428; doi:10.3389/fmicb.2016.01868)
Supplement: Supplementary file 1 [file Table_1.DOCX]

| **Table S1 Strains** | | |
| --- | --- | --- |
| **Strain Number** | **Background^a^** | **Description^b^** |
| IS75 | 168 derivative | *his leu met* |
| BD3338 | *B. subtilis* RO-OO-2 | P*comK*::*gfp* (Cm) |
| BD3339 | *B. subtilis* RO-FF-1 | P*comK*::*gfp* (Cm) |
| BD3341 | *B. subtilis* RO-E-2 | P*comK*::*gfp* (Cm) |
| BD4374 | *B. subtilis* (IS75) | P*comK*::*cfp* (Kan) |
| BD4773 | *B. subtilis* (IS75) | P*comK*::*luc* (Cm) |
| BD4893 | *B. subtilis* (IS75) | ∆*comK* (Spc), P*comK*::*luc* (Cm) |
| BD6432 | *B. subtilis* (PS216) | P*comK*::*luc* (Cm) |
| BD6434 | *B. subtilis* (PS216) | P*comK*::*cfp* (Kan) |
| BD6437 | *B. subtilis* (PS216) | ∆*comK* (Spc), P*comK*::*luc* (Cm) |
| BD6438 | *B. subtilis* (3610) | ∆*comI*, ∆*comK* (Spc), P*comK*::*luc* (Cm) |
| BD6439 | *B. subtilis* (3610) | ∆*comI*, P*comK*::*luc* (Cm) |
| BD6441 | *B. subtilis* (3610) | ∆*comI*, P*comK*::*cfp* (Kan) |
| BD7125 | *B. subtilis* (3610) | ∆*comI*, P*comG*::*luc* (Cm), ∆*degQ* (Tet) |
| BD7447 | *B. subtilis* (3610) | ∆*comI*, PcomK::*luc* (Cm), *degQ ^IS75^* |
| BD7448 | *B. subtilis* (3610) | ∆*comI*, P*comG*::*luc* (Cm), *degQ ^IS75^* |
| BD7456 | *B. subtilis* (IS75) | P*comK*::*luc* (Cm), *degQ^3610^* |
| BD7457 | *B. subtilis* (IS75) | P*comG*::*luc* (Cm), *degQ^3610^* |
| BD8276 | *B. subtilis* (IS75) | P*srfA*::*luc* (Cm), *degQ^3610^* |
| BD8277 | *B. subtilis* (3610) | ∆*comI*, PsrfA::*luc* (Cm), *degQ ^IS75^* |
| BD8278 | *B. subtilis* (IS75) | P*srfA*::*luc* (Cm), ∆*degS* (Kan) |
| BD8279 | *B. subtilis* (IS75) | P*comK*::*luc* (Cm), ∆*degS* (Kan) |
| BD8280 | *B. subtilis* (168) | P*comG::luc* (Cm), ∆*degS* (Kan) |
| BD8281 | *B. subtilis* (3610) | ∆*comI*, P*srfA*::*luc* (Cm), ∆*degS* (Kan) |
| BD8282 | *B. subtilis* (3610) | ∆*comI*, P*comK*::*luc* (Cm), ∆*degS* (Kan) |
| BD8283 | *B. subtilis* (3610) | ∆*comI*, P*comG*::luc (Cm), ∆*degS* (Kan) |
| BD8284 | *B. subtilis* (IS75) | P*srfA*::*mCherry* (Kan), P*comG*::*gfp* (Cm) |
| BD8285 | *B. subtilis* (IS75) | P*srfA*::*mCherry* (Kan), P*comG*::*gfp* (Cm), *degQ^3610^* |
| BD8286 | *B. subtilis* (3610) | ∆*comI*, P*srfA*::*mCherry* (Kan), P*comG*::*gfp* (Cm) |
| BD8287 | *B. subtilis* (3610) | ∆*comI*, P*srfA*::*mCherry* (Kan), P*comG*::*gfp* (Cm), *degQ^IS75^* |
| BD8288 | *B. subtilis* (IS75) | P*xyl*-*comK* (Ery), P*hyper-spank*-*degQ* (Kan) |
| BD8289 | *B. subtilis* (3610) | ∆*rapP*, P*srfA*::*luc* (Cm) |
| BD8290 | *B. subtilis* (3610) | ∆*rapP*, P*comK*::*luc* (Cm) |
| BD8291 | *B. subtilis* (3610) | ∆*rapP*, ∆*comK* (Spc), P*comK*::*luc* (Cm) |
| BD8292 | *B. subtilis* (3610) | ∆*comI*, P*srfA*::*luc* (Cm), *degUD56N* (Kan) |

^a^The IS75 derivatives are all *his leu met* auxotrophs. ^b^All of the fusion constructs and the P*hyper-spank-degQ* construct are inserted by single crossover at the native loci. The P*xyl-comK* construct is at the *amyE* locus.
