# Supplementary material for: A DegU-P and DegQ-Dependent Regulatory Pathway for the K-state in Bacillus subtilis
Source: Front Microbiol. 2016 Nov 22;7:1868. doi: 10.3389/fmicb.2016.01868 (PMC5118428; doi:10.3389/fmicb.2016.01868)

Figure S1 | The timing and duration of transformation is different in the domesticated strain IS75 and the two undomesticated strains 3610 and PS216. Transformation efficiency (grey) and turbidity (black) were determined every hour for 7 hours. Note that while the turbidity scale, measured in a Klett colorimeter, is the same for the three panels (left axes), the transformation efficiencies are plotted on different scales.

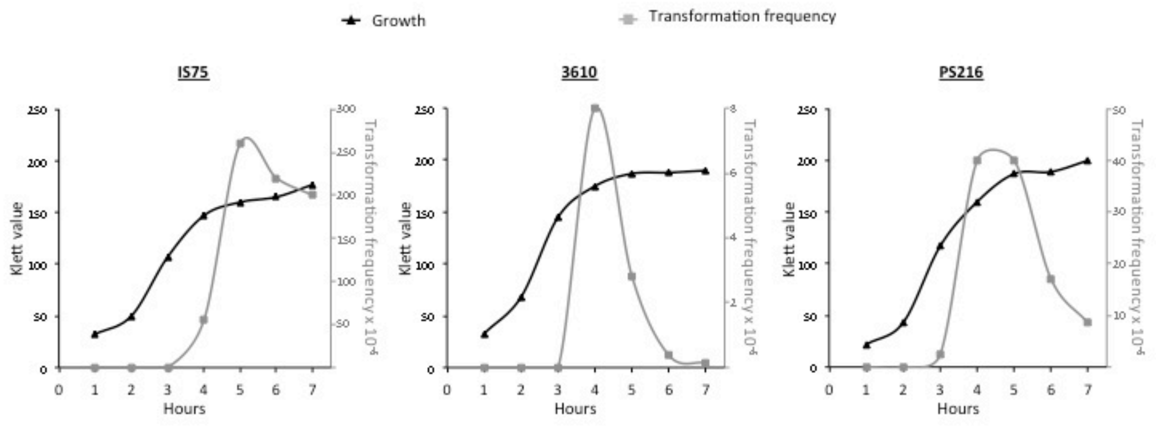

Figure S2 | Effect of a *ΔdegQ* mutation on the transcription rate from P*comG* in the 3610 background. The vertical arrow points to T_0.

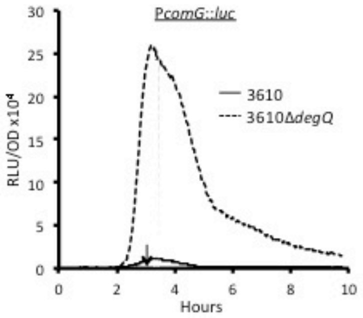

Figure S3 | Normalized curves for *srfA, comK* and *comG* transcription rates in the *degQ* swapped strains in the IS75 (A) and 3610 (B) backgrounds. To more graphically compare the shapes of the rate curves, the data from Fig. 2 has been normalized by the maximum value for each curve. The vertical arrows in each panel point to T_0.

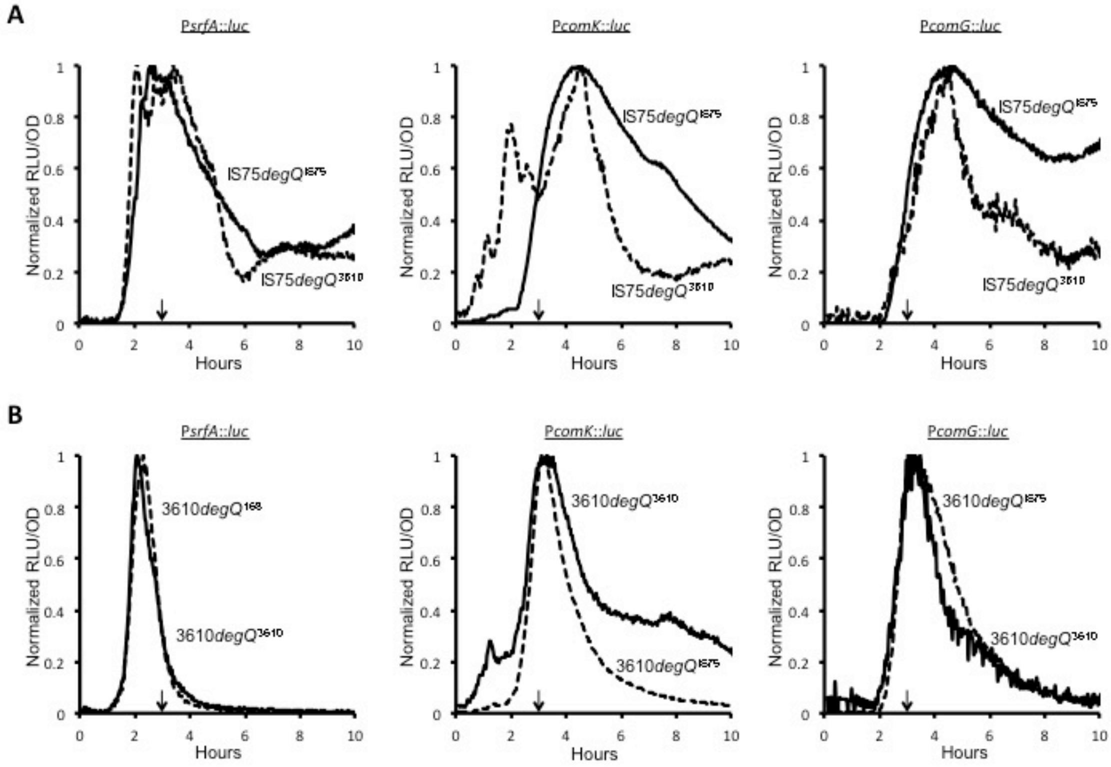

Figure S4 | Transcription rate data for P*srfA* in the IS75, PS216 and 3610 backgrounds, plotted together (A). Panel B shows the data from panel A, normalized using the maximum of each curve. In this experiment, PS216 reached T_0 earlier than the other two strains.

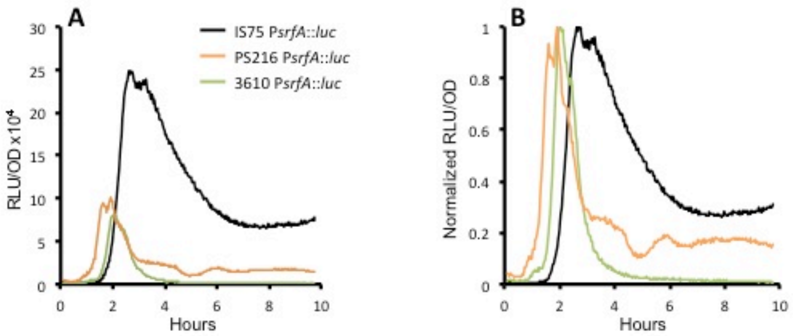

Figure S5 | Transcription rates of P*srfA-luc* in the 3610 wild-type and *degU*^{D56N} strains.

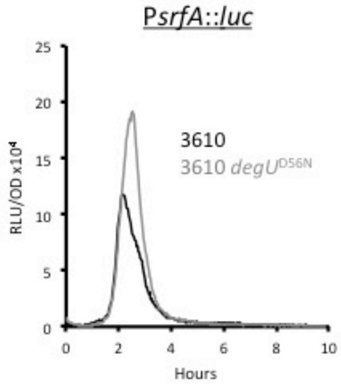

Figure S6 | Effect of the *ΔrapP* mutation on transcription rates from P*srfA*, P*comK* and P*comK* in the *ΔcomK* background.

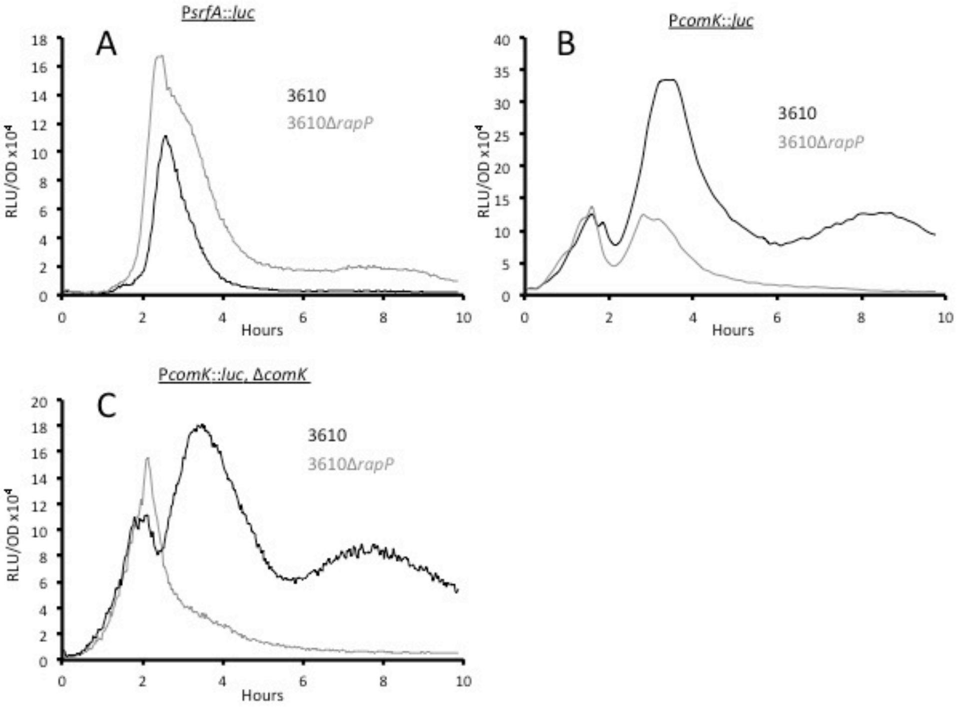

Figure S7 | The basal expression from P*comK* is comparable in IS75, PS216 and 3610. All three strains carry P*comK-luc* and *ΔcomK*.

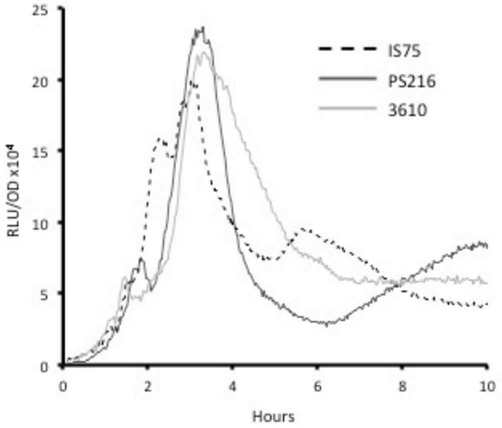

Supplement: Supplementary file 2 [file Presentation_1.PDF]
